# Antioxidant and Anti-Inflammatory Activity of *Filipendula glaberrima* Nakai Ethanolic Extract and Its Chemical Composition

**DOI:** 10.3390/molecules27144628

**Published:** 2022-07-20

**Authors:** Jaemee Jung, Hoon Kim, Sanghyun Lee, Mijin Hong, Dahyun Hwang

**Affiliations:** 1 Department of Biomedical Laboratory Science, College of Software and Digital Healthcare Convergence, Yonsei University, Wonju 26493, Korea; jmon2984@naver.com; 2 Department of Food and Nutrition, Chung-Ang University, Anseong 17546, Korea; saphead1106@hanmail.net; 3 Department of Plant Science and Technology, Chung-Ang University, Anseong 17546, Korea; slee@cau.ac.kr; 4 Department of Integrated Biomedical and Life Science, College of Health Sciences, Korea University, Anam 02841, Korea; mijiiin1216@naver.com; 5 Department of Biomedical Laboratory Science, College of Life and Health Sciences, Hoseo University, Asan 31499, Korea; 6 The Research Institute for Basic Sciences, Hoseo University, Asan 31499, Korea

**Keywords:** *Filipendula glaberrima*, antioxidant, anti-inflammatory, tandem mass spectrometry, bioactive flavonoid

## Abstract

Many countries are endeavoring to strengthen the competitiveness of their biological resources by exploring and developing wild endemic plants. This study examined the effects of *Filipendula glaberrima* Nakai (FG) on the antioxidant and anti-inflammatory activity using an in vitro system. The bioactive components were also examined using chromatographic techniques. The ethanol extract of *Filipendula glaberrima* Nakai (FGE) exerted antioxidant activities in the radical scavenging and reducing power assays and had high amounts of total polyphenolic compounds. The qRT-PCR results suggested that FGE significantly downregulated the levels *cyclooxygenase (COX)-2*, *inducible nitric oxide synthase (iNOS) 2*, *tumor necrosis factor (TNF)-α*, and *interleukin (IL)-6* in LPS-stimulated RAW 264.7 cells. The FGE treatment also decreased the production of nitric oxide, TNF-α, and IL-6 significantly in a dose-dependent manner. In addition, FGE downregulated phosphorylation of MAPK and NF-κB signaling pathway-related proteins. The chromatographic and mass spectrometry results showed that FGE contained bioactive flavonoids such as (+)-catechin, miquelianin, quercitrin, and afzelin, which may be active compounds with antioxidant and anti-inflammatory activities. This study provides fundamental data on the anti-inflammatory activity of the FG and can serve as a good starting point for developing a novel natural anti-inflammatory agent using FGE-containing bioactive flavonoids.

## 1. Introduction

Inflammation is a vital defense mechanism for protecting the human body against hazardous stimuli, such as pathogens, damaged cells, toxic compounds, or irradiation [1,2]. The inflammation reaction helps suppress cell damage in the early stages, prevent infection, remove destroyed tissue and necrotized cells from the damaged tissue, and regenerate tissue in the body [1,2,3]. Inflammation is not a disease itself but a pivotal defense system operated by the immune system. On the other hand, chronic inflammation from uncontrolled acute inflammation can affect certain parts of the body and may contribute to multiple chronic diseases, such as allergies, cardiovascular and bowel diseases, diabetes, metabolic syndrome, arthritis, cancer, and autoimmune diseases [1,4]. Although a variety of purified or synthetic drugs have been developed for regulating and alleviating inflammatory responses, such as steroids, non-steroidal anti-inflammatory drugs (NSAIDs), and immunosuppressants, their clinical applications are strictly limited to the minimum dose that exerts the least adverse effects because of their several unwanted side effects, including potential toxicity [4,5]. By contrast, various edible and medicinal plants have been considered favorable resources for alleviating and treating inflammatory reactions [4,6]. Thus, current interests in exploring and developing anti-inflammatory agents have focused on natural resources, such as herbal and medicinal plants, to increase their efficacy and reduce side effects [5].

The Nagoya Protocol on Access and Benefit Sharing (ABS), which was a supplementary agreement based on the Convention on Biological Diversity (CBD), was adopted in 2010 for the fair and equitable sharing of benefits arising from genetic resources [7,8]. Many countries are endeavoring to strengthen the competitiveness of their biological resources by exploring and developing wild endemic plants [9]. Korea has more than 100,000 relatively diverse native species [8]. According to the Korean Government, the number of native species increased to 41,483 at the end of 2013. Among them, 2177 species are endemic plants, with an estimated 38,000 indigenous species [8].

The genus *Filipendula* is a perennial herbaceous plant in the family Rosaceae that includes approximately 20 species and grows mainly in the temperate regions of the Northern hemisphere, Eastern Siberia, and Northeast Asia, including Korea, Japan, and Manchuria [10,11]. Most *Filipendula* species generally grow in the high mountains with humid and shady areas. *Filipendula glaberrima* Nakai (FG), known as Korean meadowsweet, including subspecies such as *F. formosa* and *F. koreana* [10,12]. Wild FG has been used traditionally to treat inflammation, pain, and gout, but FG has attracted less research attention than other *Filipendula* species. There are few physiological and pharmacological studies on FG so far, and some published papers are related to its chemical characteristics mainly including bioactive compounds [10,13]. Moreover, there are no reports on using the aerial parts of FG rather than other tissues, such as stems and roots. This may be due to the rarity of FG arising from difficult cultivation characteristics in high mountainous surroundings. Thus, an investigation of the chemical and physiological properties and the establishment of cultivation methods are needed to strengthen the competitiveness of functional raw materials using endemic plants [14].

This study investigated the effect of the ethanol extract of *Filipendula glaberrima* Nakai (FGE) on antioxidant and anti-inflammatory activity using the in vitro cell culture system and identified its bioactive components using chromatographic techniques.

## 2. Results

### 2.1. Antioxidant Capacities and Polyphenolic Content of FGE

The antioxidant activity of FGE was determined by measuring the radical scavenging activities, reducing power, and total polyphenolic contents, as listed in Table 1. The scavenging activities of FGE against 2,2-azino-bis(3-ethylbenzothiazoline-6-sulfonic acid) (ABTS) and 2,2-diphenyl-1-picrylhydrazyl (DPPH) radicals showed IC_50_ values of 421.2 and 524.6 µg/mL, respectively, which corresponded to 2.1 and 4.1 times higher than ascorbic acid (200.0 and 128.2 µg/mL, respectively) that was used as a positive control. The result of reducing power using the ferric reducing antioxidant power (FRAP) method showed that FGE contained 221.2 mmole FSE/g, which was approximately 5.2-fold lower than that of ascorbic acid (1150.3 µmole FSE/g). The polyphenolic content in FGE was next measured to estimate the concentrations of the compounds that exert antioxidant activity. The result suggested that FGE has high total polyphenolic compounds by 214.4 mg GAE/g.

### 2.2. Cytotoxic Effect and the Morphological Alteration Induced by FGE in lipopolysaccharide (LPS)-Stimulated RAW 264.7 Cells

**Figure 1:**
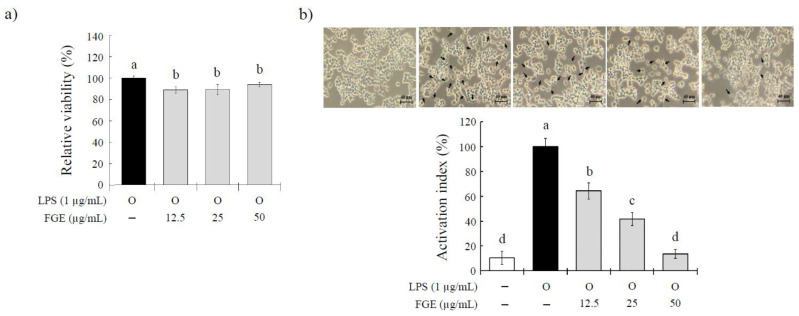
Effect of *Filipendula glaberrima* Nakai extract on the morphological changes and cytotoxicity on LPS-treated RAW 264.7 macrophages. RAW 264.7 cells were treated with each sample for 30 min, and then LPS (1 µg/mL) was stimulated for a further 24 h. (**a**) The cell viability was measured using a commercial MTT assay. (**b**) The cell morphology was photographed using a microscope, and dendritic-like cells (black arrows) were counted to calculate the activation index of the cells. The results are expressed as the mean ± S.D. of three independent tests in triplicate. Different superscripts mean a significant difference among groups (*p* < 0.05) based on one-way ANOVA and Duncan’s multiple range test. LPS, lipopolysaccharide; FGE, ethanol extract of *Filipendula glaberrima* Nakai.

The cytotoxic effects of FGE against LPS-stimulated RAW 264.7 cells were determined using a conventional MTT assay (Figure 1a). Compared to the LPS-treated control cells, cell viability was decreased significantly in the cells treated with FGE by 5.4–16.2% at doses of 12.5–50 μg/mL. On the other hand, all doses of FGE induced survival rates >80%, which are considered non-cytotoxicity. The macrophage activation index was measured by evaluating the morphological modifications on LPS-treated RAW 264.7 cells. These results are provided as morphological images of the cells and their quantified results. As shown in Figure 1b, the LPS treatment induced significant morphological changes from the spherical shape into a dendritic-like shape than normal cells, suggesting evidence of activation. Compared to the LPS-treated group, the dendritic-like shapes were significantly lower (by 35.7–86.3%) in the FGE-treated groups in a dose-dependent manner, suggesting that the activation of the cells induced by LPS stimulation was inhibited by the FGE treatment.

### 2.3. Effect of FGE Treatment on the Inhibition of Pro-Inflammatory Genes and Proteins in LPS-Treated RAW 264.7 Cells

**Figure 2:**
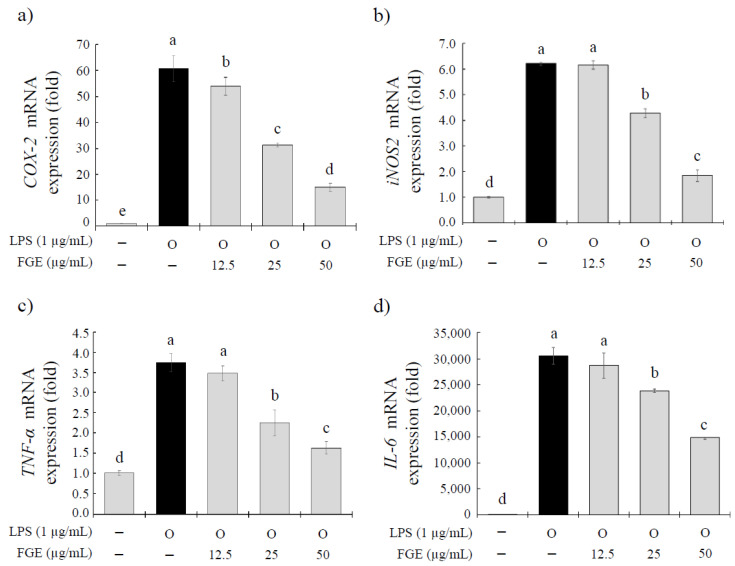
Effect of *Filipendula glaberrima* Nakai extract on the expression of (**a**) *COX-2*, (**b**) *iNOS2*, (**c**) *TNF-α*, and (**d**) *IL-6* genes in LPS-treated RAW 264.7 macrophages. RAW 264.7 cells were treated with each sample for 30 min, and then LPS (1 µg/mL) was stimulated for a further 24 h. The mRNA expressions were analyzed by qRT-PCR. The results are expressed as the mean ± S.D. of three independent tests in triplicate. Different superscripts mean a significant difference among the groups (*p* < 0.05) based on one-way ANOVA and Duncan’s multiple range test. FGE, ethanol extract of *Filipendula glaberrima* Nakai; *COX-2*, *cyclooxygenase-2*; *iNOS2*, *inducible nitric oxide synthase 2*; *TNF-α*, *tumor necrosis factor-alpha*; *IL-6*, *interleukin-6*.

The inhibitory activity against proinflammatory mediators was measured at the gene and protein levels to determine if FGE affects anti-inflammatory activity. Figure 2 shows the mRNA expression of the proinflammatory mediators, including *cyclooxygenase (COX)-2* (Figure 2a), *inducible nitric oxide synthase (iNOS)2* (Figure 2b), *tumor necrosis factor (TNF)-α* (Figure 2c), and *interleukin (IL)-6* (Figure 2d) analyzed by qRT-PCR. Compared to the LPS-treated control, FGE-treated groups exhibited significantly decreased levels of all genes in a dose-dependent manner. The FGE treatment downregulated the *COX-2* gene expression significantly at all doses tested (12.5–50 μg/mL, 11.2–76.5% inhibition). By contrast, the significantly lower expressions of *iNOS* (37.2 and 83.8% inhibition), *TNF-α* (54.3 and 77.2% inhibition), and *IL-6* (22.0 and 51.3% inhibition) genes were observed at doses of only 25 and 50 μg/mL, compared to the LPS-treated control. These results suggest that the FGE treatment inhibited the expression of these proinflammatory genes.

**Figure 3:**
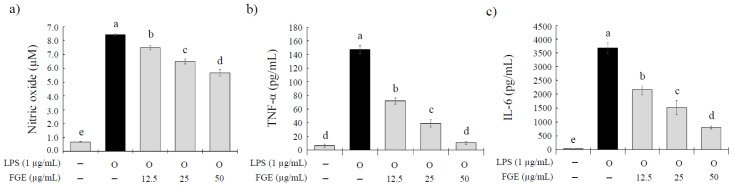
Effect of *Filipendula glaberrima* Nakai extract on the production of NO, TNF-α, and IL-6 protein expression in LPS-treated RAW 264.7 cells. RAW 264.7 cells were treated with each sample for 30 min, and then stimulated with LPS (1 µg/mL) for a further 24 h. The production of (**a**) NO, (**b**) TNF-α, and (**c**) IL-6 were analyzed using a Griess assay and ELISA method, respectively. The results are expressed as mean ± S.D. of three independent tests in triplicate. Different superscripts mean a significant difference among groups (*p* < 0.05) based on a one-way ANOVA and Duncan’s multiple range test. FGE, ethanol extract of *Filipendula glaberrima* Nakai; NO, nitric oxide; TNF-α, tumor necrosis factor-alpha; IL-6, interleukin-6.

The above results show that the FGE treatment inhibited the expression of these proinflammatory genes. Therefore, this study further examined whether FGE inhibits the secretion of these proinflammatory mediators. The effects of the FGE treatment on the secretion of nitric oxide (NO), TNF-α, and IL-6 in LPS-stimulated RAW 264.7 cells were analyzed by ELISA, as shown in Figure 3. At all doses tested (12.5–50 μg/mL), the FGE treatment decreased the secretion of NO, TNF-α, and IL-6 significantly in a dose-dependent manner. The inhibitory rates were 12.0–35.4% for NO, 53.5–96.9% for TNF-α, and 41.6–78.9% for IL-6.

### 2.4. Effect of FGE Treatment on the Phosphorylation of MAPKs and NF-κB in LPS-Treated RAW 264.7 Cells

**Figure 4:**
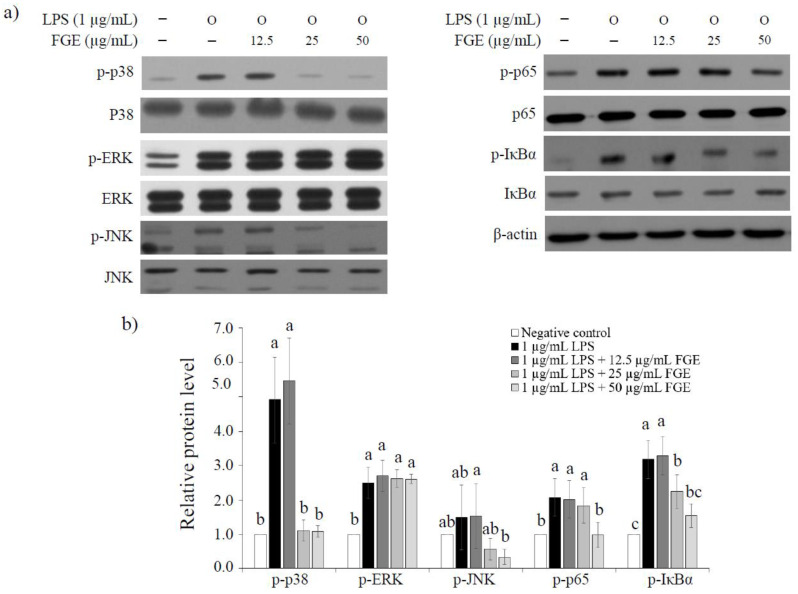
Effect of *Filipendula glaberrima* Nakai extract on the phosphorylation of MAPKs and NF-κB in LPS-treated RAW 264.7 cells. RAW 264.7 cells were treated with each sample for 30 min, and then stimulated with LPS (1 µg/mL) for a further 30 min. (**a**) Protein was extracted and immunoblotted with the indicated antibodies. β-actin was used as internal loading control. (**b**) Data analysis was performed using ImageJ software by measuring the integrated band densities following background subtraction. The results are expressed as mean ± S.D. of three independent tests in triplicate. Different superscripts mean a significant difference among groups (*p* < 0.05) based on a one-way ANOVA and Duncan’s multiple range test. FGE, ethanol extract of *Filipendula glaberrima* Nakai; MAPK, mitogen-activated protein kinase; NF-κB, nuclear factor kappa B; LPS, lipopolysaccharide; p-p38, phospho-p38; p-ERK, phospho-ERK; ERK, extracellular signal-regulated kinase; p-JNK, phospho-JNK; JNK, Jun N-terminal kinase; p-p65, phospho-p65; p-IκBα, phospho-IκBα; IκBα, nuclear factor of kappa light polypeptide gene enhancer in B-cells inhibitor, alpha; β-actin, beta actin.

To investigate the signaling pathway by FGE, we measured the degree of phosphorylation of mitogen-activated protein kinase (MAPK) (p38, ERK, JNK) and nuclear factor kappa B (NF-κB) (p65, IκBα) by treating macrophages with various concentrations of FGE for 30 min. Compared to the LPS-treated control cells, p-38 and p-JNK were significantly restrained phosphorylation at 77.9% and 77.8% at doses of 50 μg/mL FGE, respectively (Figure 4). However, phosphorylation of ERK was not induced by FGE treatment. Next, we investigated the effect of FGE on the NF-κB pathway mediated by p65 and IκBα. FGE significantly increased p65 phosphorylation-inhibition and IκBα degradation at 52.5% and 51.6% compared to the LPS-treated control cells at the treatment of 50 μg/mL. In addition, the total protein content of JNK, p38, ERK, p65, and IκBα was not affected by FGE treatment. This indicates that anti-inflammatory activity by FGE is related to the activation of MAPK and NF-κB signaling pathways in macrophages.

### 2.5. Identification and Quantification of the Major Components of FGE

**Figure 5:**
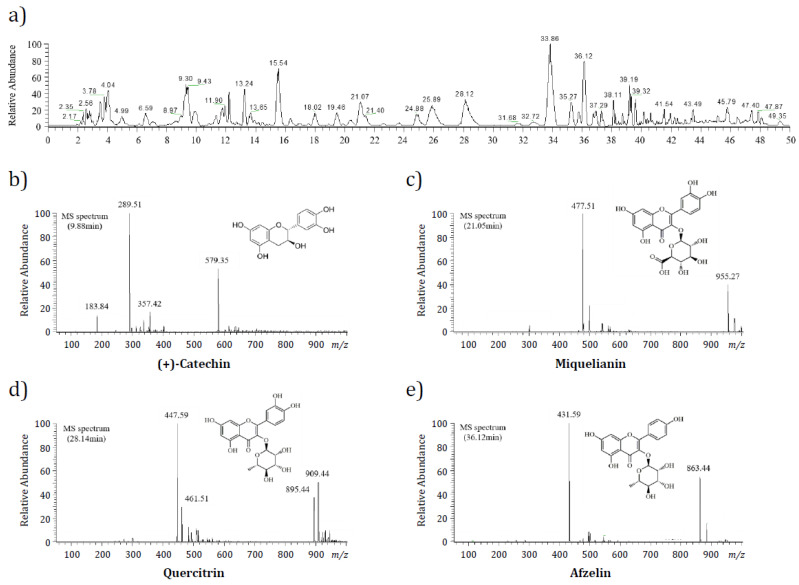
UHPLC-MS chromatogram and mass spectrum of *Filipendula glaberrima* Nakai extract. (**a**) Total ion chromatogram. (**b**–**e**) Mass spectra of the peaks at 9.88 min, 21.05 min, 28.14 min, and 36.12 min on the total ion chromatogram. A 30 mg/mL of FGE was introduced to UHPLC-MS for analysis.

**Figure 6:**
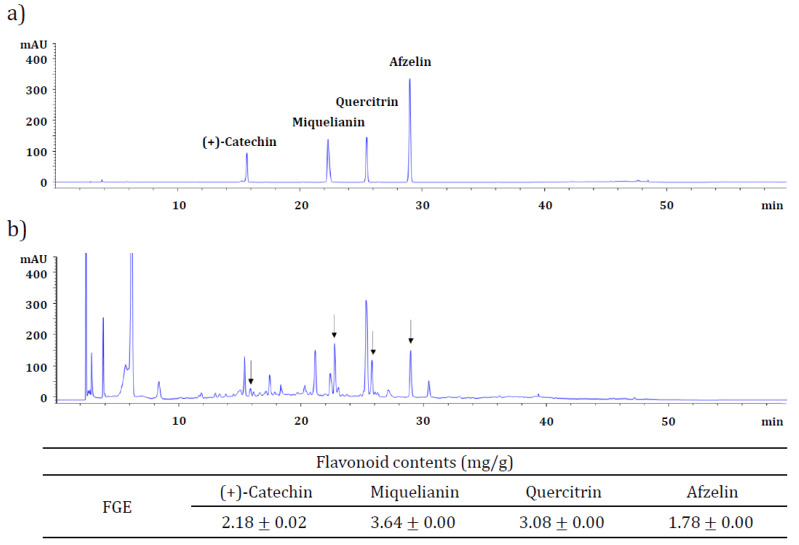
(**a**) HPLC chromatogram of a mixture of four flavonoid references comprising (+)-catechin, miquelianin, quercitrin, and afzelin (250 μg/mL each). (**b**) HPLC chromatogram of FGE (1 mg/mL).

FGE was analyzed by ultra-high-performance liquid chromatography-mass spectrometry (UHPLC-MS) and high-performance liquid chromatography-diode array detector (HPLC-DAD) to identify and quantify the major active ingredients. UHPLC equipped with ESI and MS was introduced to estimate the major compounds in FGE. The resulting PDA and full MS chromatograms were sorted manually to search the information based on the retention time, *m*/*z* values, and MS fragmentation patterns. Each peak in the MS spectrum was analyzed using the natural-product database available online and in-house library. From the full MS chromatogram (total ion chromatogram) of FGE, the four peaks positioned at 9.88, 21.05, 28.14, and 36.12 min were analyzed further by MS spectrometry with negative ion mode, as shown in Figure 5a (Figure 5b–d). Based on the spectral library, the ion peak at 9.88 min on the full MS chromatogram had a major molecular weight of *m*/*z* 289.51 ([M‒H]^−^) as the base peak, suggesting the existence of flavonoid (+)-catechin (CAS No. 154-23-4; Figure 5b). Simultaneously, other peaks at 21.05, 28.14, and 36.12 min on the full MS chromatogram showed base peaks of *m*/*z* 477.51 ([M‒H]^−^), 447.59 ([M‒H]^−^), and 431.59 ([M‒H]^−^), respectively, which were estimated to be three flavonoid glycosides, miquelianin (quercetin 3-*O*-glucuronide; Figure 5c), quercitrin (quercetin 3-*O*-rhamnoside; Figure 5d), and afzelin (kaempferol 3-*O*-rhamnoside; Figure 5e). Next, these four putative compounds were identified by HPLC-DAD. The chromatographic fingerprinting method was carried out to compare the retention time of peaks between the commercial references and FGE. Figure 5b and Figure 6a show the HPLC chromatograms obtained from the standard mixture comprising the four flavonoid references and FGE, respectively. The four peaks shown in Figure 6a (standard chromatogram) were identified clearly in the chromatogram obtained from FGE (Figure 6b), indicating that FGE contained the four flavonoids. Finally, the calibration curve of the four flavonoid references was obtained using an external standard method (data not shown), using five concentrations of the standard mixtures, with three injections per concentration. FGE contained 2.18 mg/g, 3.64 mg/g, 3.08 mg/g, and 1.78 mg/g (+)-catechin, miquelianin, quercitrin, and afzelin, respectively.

## 3. Discussion

The localization of biological resources is required after the Nagoya Protocol on ABS was applied in 2014. Therefore, there is an increasing number of studies on developing functional ingredients from natural substances considered safe and with fewer adverse effects than synthetic materials [4,15,16]. The present study evaluated FGE as a plant with anti-inflammatory properties. The results showed that compared to ascorbic acid used as the positive control, FGE showed a 2.1 to 4.1-fold lower scavenging activity against ABTS and DPPH free radicals, respectively. FGE also showed a 5.2-fold lower FRAP activity than ascorbic acid. Given that ascorbic acid is one of the most efficacious antioxidants and is manufactured commercially as a pure compound, the results suggested that FGE, which was a crude mixture comprised of a variety of compounds, can be considered a predominant natural antioxidant candidate. Several studies reported the antioxidant activity of extracts prepared from different species of the *Filipendula* family, particularly *F. ulmaria* (common meadowsweet) [17,18,19]. On the other hand, this is the first to show the antioxidant activity from *F. glaberrima* Nakai (Korean meadowsweet). The antioxidant capacity of plants depends mainly on the compositions of various polyphenolic compounds with different mechanisms of action [20]. These polyphenols are closely involved in many health benefits via their antioxidant actions. Hence, this study examined the contents of polyphenolic compounds in FGE. The results showed that the total polyphenol contents of FGE were approximately 214.4 GAE/g (21.4 w/w%). Therefore, FGE may be used to develop potential natural medications with considerable therapeutic values against various diseases related to the generation of free radicals, including inflammation. This is because oxidative stresses caused by imbalanced metabolisms within the cells and tissues can cause many chronic inflammatory diseases [21], and plant-derived antioxidants, such as polyphenols, flavonoids, carotenoids, coumarins, lignans, and terpenoids, are generally considered meaningful as an adjuvant therapy because of their role in alleviating inflammatory reaction [22,23].

The inflammatory process is usually controlled by signals involving initiate/maintain and shut down inflammation processes [24], but unbalanced acute inflammation caused by infections, pathogens, external injuries, harmful chemicals, and radiation may cause chronic inflammation, which can provoke chronic inflammatory diseases, including heart diseases, rheumatoid arthritis, diabetes, obesity, asthma, dementia, stroke, and cancer [1,3,24,25]. Macrophages are the most important immune cells in various immune responses, particularly those crucial in initiating, maintaining, and recovering during the inflammation process [24,26,27]. After stimulation by activation signals, including bacterial LPS, extracellular matrix proteins, cytokines, and external chemical mediators, macrophages initiate an inflammation process by secreting proinflammatory cytokines, such as IL-1, IL-6, IL-8, IL-12, and TNF-α, and oxidative mediators, such as NO and reactive oxygen species (ROS) [28,29]. In addition, prostaglandins (PGEs) are important activators in the inflammatory response [30]. Therefore, an evaluation of these proinflammatory mediators in activated macrophages has been used to develop anti-inflammatory agents [27]. These results show that both genes (*COX-2*, *iNOS*, *TNF-α*, and *IL-6*) and the corresponding proinflammatory mediators (NO, TNF-α, and IL-6) were decreased dose-dependently by the FGE treatment in LPS-stimulated RAW 264.7 cells. The effective doses of FGE in exerting anti-inflammatory activity were only 12.5–50 µg/mL.

The MAPK and NF-κB signaling pathways in RAW264.7 cells are well-known immune activation mechanisms [31,32]. In particular, plant-derived polysaccharides are known to activate intracellular signaling pathways, such as MAPK and NF-Κb, in combination with pattern recognition receptors in macrophages [33]. The MAPK pathway is mediated by extracellular signaling kinases (that is to say p38, ERK, and JNK) and is regulated through the phosphorylation of these enzymes [34]. Of these, the ERK pathway is induced generally by mitotic and proliferative stimuli, whereas the p38 and JNK pathways are activated by inflammation and external physical damage [35]. The transcription factor NF-κB regulates several aspects of innate and adaptive immunity and participates in inflammasome regulation by inducing the expression of proinflammatory genes [36]. NF-κB activity is maintained by the movement of the p65 and IκBα complexes between the cytoplasm and the nucleus [37]. The IκBα proteins bind to p65, masking their nuclear localization signal and causing their cytoplasmic retention [37]. Whether FGE is involved in phosphorylation of these two pathways was analyzed for representative signaling pathways (MAPK and NF-κB) of macrophages. In present study, we demonstrated that FGE significantly phosphorylated MAPK (p-p38 and p-JNK) and NF-κB (p-p65 and IκBα) in a concentration-dependent manner. Therefore, FGE is assumed to be involved in anti-inflammatory by regulating the expression of inflammatory cytokines through inhibition of MAPK/NF-κB signaling cassettes. To the best of the authors’ knowledge, this is the first study to show that the *F*. *glaberrima* extract can exert anti-inflammatory activity by inhibiting the proinflammatory genes and mediators, and can be utilized as a natural anti-inflammatory candidate. On the other hand, further studies will be needed to identify its intracellular mechanism of action underlying anti-inflammatory activity and increase the possibility of industrial use of FGE, which will be performed in future work.

UHPLC-MS confirmed that four flavonoids were contained in FGE, which were all classified as the flavonoid family: (+)-catechin, miquelianin (quercetin 3-*O*-glucuronide), quercitrin (quercetin 3-*O*-rhamnoside), and afzelin (kaempferol 3-*O*-rhamnoside). HPLC-DAD analysis showed that these flavonoids were present in FGE, and the (+)-catechin, miquelianin, quercitrin, and afzelin contents were 2.18 mg/g, 3.64 mg/g, 3.08 mg/g, and 1.78 mg/g, respectively. Flavonoids and their derivatives (mainly flavonoid glycosides) have been used for centuries to prevent and treat various disorders, including chronic inflammatory diseases [25]. A variety of biological properties, such as antioxidant, anticancer, antibacterial, anti-inflammation, and anti-allergy of the flavonoid family, including catechin, quercetin, kaempferol, and their derivatives, have been reported [25,38,39,40]. Therefore, despite their low amounts, these fundamental flavonoids can play an important role in exerting antioxidant and anti-inflammatory activity in FGE. However, to verify the main active component in FGE clearly, further investigations are required. Most studies on the characterization and identification of the major and active constituents of *Filipendula* species focused on *F. ulmaria* [17,18,19,41]. On the other hand, few studies have been conducted to determine the bioactive constituents from FG. The present study provides fundamental data on the anti-inflammatory activity of FG and suggests that bioactive flavonoids, including catechin, miquelianin, quercitrin, and afzelin may be active compounds in exerting anti-inflammatory activity of FGE. In addition, this study can be a good starting point for developing a novel natural anti-inflammatory agent using FGE-containing bioactive flavonoids.

## 4. Materials and Methods

### 4.1. Sample Preparation

Whole tissue of wild FG plant was obtained from the demilitarized zone, adjacent to northern Gyeonggi province (Yeoncheon-gun, Korea). The plant was identified by Dr. Jin-Kyu Kim, a senior researcher at Gyeonggido Business and Science Accelerator, Gyeonggi Biocenter (Suwon, Korea). A voucher specimen (No. GB-0213) was deposited in the same department described above. FG used upper-layer plants (stems, leaves, flowers) except for the roots. The upper part in contact with air was used except for the root part of FG. The aerial parts of FG (55.5 g) were extracted twice with 70% aqueous ethanol (1 L) at room temperature for two days and named FGE. The combined extracts were filtrated (Advantec No.2) and concentrated in vacuo at 40 °C (EYELA rotary evaporator, Tokyo, Japan) and freeze-dried to yield 7.55 g of the residue. The dried extract was dissolved in DMSO at 100 mg/mL and diluted appropriately with distilled water for the experiments.

### 4.2. Total Polyphenol Content Determination

The Folin-Ciocalteu method [42] was used to measure the total polyphenol content of FGE. The extract was diluted to the required concentration, and the same amount of 2% Na_2_CO_3_ was added. After leaving the mixed solution to stand for 3 min, 200 μL of 50% Folin-Ciocalteu reagent was added, and the mixture was stirred for 10 s using a plate shaker. After reacting in a dark room for 30 min, the absorbance was measured using a UV/Visible spectrophotometer at 750 nm. The concentration was calculated from a standard curve using gallic acid. The total polyphenol content was determined from the final gallic acid concentration set to 0–500 μg/mL.

### 4.3. Measurement of FRAP

The reducing power of the FGE extract was measured using the FRAP assay [43]. To prepare the FRAP reagent, 200 mL of 300 mM acetate buffer (pH 3.6) was mixed with 20 mL of 10 mM 2,4,6-tripyfidyl-s-triazine (TPTZ), 320 mL of 20 mM FeCl_3_, and 20 mL of DIW. After adding 200 μL of the mixed FRAP reagent and 10 μL of the extract and reacting for 4 min, the absorbance was measured at 593 nm. As a standard substance, iron (II) sulfate heptahydrate was used for measurement in the same manner as the sample. The reducing power of the sample was calculated from a standard calibration curve.

### 4.4. Radical Scavenging Activity

The antioxidant activity of the extract was measured using the ABTS free radical scavenging assay [44]. The ABTS stock solution was prepared by reacting 7.4 mM ABTS and 2.6 mM potassium persulfate in a dark room at 4 °C for 24 h. The stock was diluted 100 times in distilled water to make ABTS working solution. Subsequently, 200 μL of the diluted working solution and 10 μL of the extract were added and reacted in a dark room for 30 min. The absorbance was measured at 734 nm. The antioxidant activity of the extract was measured using DPPH free radical scavenging [45]. The 0.2 mM DPPH was reacted at 4 °C for the preparation of the DPPH stock solution. The solution was diluted with methanol at 514 nm so that the absorbance was approximately 0.8. The 200 μL of diluted DPPH working solution and 10 μL of extract were added, reacted in a dark room for 30 min, and the absorbance was measured at 514 nm. As the standard substance, ascorbic acid was measured in the same manner as the sample, and the free radical scavenging of the sample corresponding to the standard calibration curve was determined.

### 4.5. Cytotoxicity Evaluation and Cell Morphology Observation

Mouse macrophages RAW264.7 cells were purchased from Korea Cell Line Bank (Seoul, Korea). The cells were cultured in DMEM (HyClone, San Angelo, TX, USA) medium supplemented with 10% fetal bovine serum (FBS; Life Technologies, Grand Island, NY, USA) and 1% penicillin-streptomycin (P/S; GenDEPOT, Katy, TX, USA) in a 37 °C incubator with 5% CO_2_. The cells were treated with FGE (12.5 to 50 μg/mL) for 30 min, and 1 μg/mL of LPS was co-treated for 24 h. To measure the cytotoxicity, the supernatants were removed, and 100 μL of 10% EZ cytox (DoGenBio, Seoul, Korea) dissolved in serum-free media was added. After 30 min, the absorbance was measured at 450 nm. The relative cell viability (%) is expressed as a percentage relative to the LPS group. The cell morphology was observed using an inverted microscope (CKX53, OLYMPUS, Tokyo, Japan). The activation index percentage is expressed as the number of cells with an activated morphology relative to the total number of cells, quantified in five random fields.

### 4.6. Determination of NO and Proinflammatory Cytokines

The RAW 264.7 macrophages were cultured overnight in 96-well plates (1 × 10^6^ cells/mL) in DMEM-FBS The cells were treated with FGE (12.5 to 50 μg/mL) for 30 min, and co-treated with 1 μg/mL of LPS for 24 h. The supernatants were collected, and the NO level was measured using the Griess reagent. In total, 50 μL of supernatant and 50 μL of Griess reagent (sulfanilamide + 85% phosphoric acid + N- (1-Naphthyl) polyethylenediamide dihydrochloride) were mixed and reacted for 10 min. The absorbance was measured at 540 nm. Sodium nitrite was used as a standard substance. The contents of TNF-α and IL-6, which are the inflammatory cytokines released in the supernatant, were analyzed by the ELISA method using a sandwich ELISA set (BD bioscience, Vancouver, Canada) according to the manufacturer’s instructions.

### 4.7. Quantitative Real-Time Polymerase Chain Reaction (qRT-PCR)

The RAW 264.7 macrophages were cultured overnight in 24-well plates (1 × 10^6^ cells/mL) in DMEM-FBS. The cells were treated with FGE (12.5 to 50 μg/mL) for 30 min, and co-treated with 1 μg/mL of LPS for 24 h. The pellets were collected, and RNA was separated using Trizol (Macherey-Nagel, Düren, Germany). The isolated RNA was reverse transcribed using a ReverTra AceTM qPCR RT Master Mix (TOYOBO, Osaka, Japan). qPCR was performed by SYBR^®^ Green Realtime PCR Master Mix reagent (TOYOBO, Osaka, Japan) using Magnetic Induction Cycler PCR Machine (biomolecular systems, Coomera, Australia). The primer sequence was as follows: *GAPDH* (Sense: 5′- ATG GTG AAG GTC GGT GTG AAC -3′, Antisense: 5′- TTG ATG TTA GTG GGG TCT CGC-3′); *COX-2* (Sense: 5′- CCC AGA GCT CCT TTT CAA CC-3′, Antisense: 5′- ATT TGG CAC ATT TGT TCC CC -3′); *iNOS2* (Sense: 5′- GCG CTC TAG TGA AGC AAA GC -3′, Antisense: 5′- TGA TGG ACC CCA AGC AAG AC-3′); *TNF-α* (Sense: 5′- AGC ACA GAA AGC ATG ATC CG-3′, Antisense: 5′- GTT TGC TAC GAC TG GGC TA -3′); and *IL-6* (Sense: 5′- CGA TGA TGC ACT TGC AGA AA -3′, Antisense: 5′- TGG AAA TTG GGG TAG GAA GG -3′)**.** The normalized expression change was expressed as the *GAPDH* control was set to 1 using the 2^−ΔΔCt^ method [46].

### 4.8. Immunoblotting

The RAW 264.7 macrophages were cultured overnight in a 60 Π dish (1 × 106 cells/mL) in DMEM-FBS. The cells were treated with FGE (12.5 to 50 μg/mL) for 30 min, and co-treated with 1 μg/mL of LPS for 30 min. the cells were washed with PBS and lysed to extract the proteins using RIPA buffer supplemented with 1 mM DTT, 10 mM β-glycerophosphate, 1 mM sodium orthovanadate, 1 mM PMSF, and a protease inhibitor cocktail (Roche, Mannheim, Germany). Protein concentration was quantified using BCA Protein Assay Kit (Thermo Scientific, Rockford, Illinois, USA). The equal amount of protein (20 μg) was electrophoresed by 10% SDS-PAGE, and transferred to an Immobilon-P PVDF membrane. The membrane was blocked with 5% BSA in TBS containing 0.1% Tween-20 (PBS-T) for 2 h at room temperature and then washed with TBS-T three times. The specific primary antibodies were added, and the membranes were incubated overnight at 4 °C with shaking. The primary antibodies were as follows: p-p38(9211, Cell signaling technology); p38(9212, Cell signaling technology); p-ERK(9101, Cell signaling technology); ERK(9102, Cell signaling technology); p-JNK(9251, Cell signaling technology); JNK(9258, Cell signaling technology); p-p65(3033, Cell signaling technology); p65(8242, Cell signaling technology); p-IκBα(2859, Cell signaling technology); IκBα(4812, Cell signaling technology); β-actin(sc-47778, santa cruz biotechnology). The membranes were washed with TBS-T three times, and incubated with horseradish peroxidase (HRP)-conjugated secondary antibody for 1 h. The membranes were visualized with the ECL system (Advansta, San Jose, CA, USA) using a gel imaging system (C400, Azure biosystems, Dublin, OH, USA).

### 4.9. Qualitative Analysis of FGE Extracts Using UHPLC-MS

HPLC-MS analysis was carried out to identify the major components in FGE. An INNO C18 Column (150 × 4.6 mm, 5 μm, YoungJin Biochrom Co., Ltd., Seongnam, Korea) and a mixed eluent composed of (A) water containing 0.1% formic acid and (B) acetonitrile containing 0.1% formic acid were used as the stationary and mobile phases, respectively. The mobile phase was programmed as follow: 0 min → 5 min (10% B), 5 min → 10 min (10% B → 17% B), 10 min → 30 min (17% B → 19% B), 30 min → 40 min (19% B → 95% B), 40 min → 45 min (95% B → 95% B), 45 min → 50 min (95% B → 10% B), and 50 min → 60 min (10% B → 10% B). The flow rate and injection volume of the sample were 1.2 mL/min and 20 μL, respectively. A heated electrospray ionization probe (250 °C) with a spray voltage of 5.0 kV was used as the ionized source, and the flow rate of sheath gas (N_2_) was set to 35 arb. A photodiode array (PDA) detector (Accela PDA 80 Hz; Thermo Scientific, Waltham, MA, USA) was scanned at a range of 100 to 800 nm, and MS (LTQ-Velos; Thermo Scientific, Waltham, MA, USA) was monitored at an *m*/*z* ratio of 100–1000. The data-dependent MS experiments were controlled using menu-driven software with the Xcalibur program (Thermo Electron Corporation, Waltham, MA, USA). All experiments were conducted under automatic gain-control conditions.

### 4.10. Quantitative Analysis of Flavonoid Using HPLC-DAD

For quantitative analysis of the major ingredients in FGE, an HPLC system (1260 Infinity II Quaternary Pump, Agilent Technologies, California, USA) coupled with an INNO C18 Column (150 × 4.6 mm, 5 μm, YoungJin Biochrom Co., Ltd., Seongnam, Korea) and a diode array detector (1260 Infinity II DAD WR, Agilent Technologies, CA, USA) at a wavelength of 270 nm was used. The column temperature and injection volume were set at room temperature and 10 μL, respectively. The gradient program for the mobile phase combined with (A) 0.25% acetic acid/water and (B) acetonitrile was as follows: 0 min → 5 min (10% B), 5 min → 15 min (10% B → 25% B), 15 min → 30 min (25% B → 35% B), 30 min → 35 min (35% B → 50% B), 35 min → 40 min (50% B → 100% B), 40 min → 45 min (100% B → 100% B), 45 min → 50 min (100% B → 10% B). The initial mobile phase condition was equilibrated for 10 min to ensure the reproducibility of the analysis. The four main components of the FGE were quantified by the calibration curve obtained from the external standard method using reference materials, such as (+)-catechin (CAS No. 154-23-4), miquelianin (CAS No. 22688-79-5), quercitrin (CAS No. 522-12-3), and afzelin (CAS No. 482-39-3). All reference materials were purchased from the Natural Product Institute of Science and Technology (Anseong, Korea).

### 4.11. Statistical Analysis

All statistical analyses were performed using SPSS version 12.0 for Windows (SPSS, Chicago, IL, USA). The values are expressed as the mean ± SD (standard deviation) of three independent experiments performed in triplicate. The statistical significance of the differences was determined using a Student’s *t*-test. A *p*-value of <0.05 was considered significant. The statistically significant differences among groups were evaluated by an analysis of variance (ANOVA), followed by Duncan’s multiple range tests. A *p*-value < 0.05 was considered significant.

## Figures and Tables

**Table 1:** Antioxidant activities and total polyphenol content of *Filipendula glaberrima* Nakai extract.

Sample	Antioxidant Activity			Total Polyphenol Content (mg GAE^3^/g)
	ABTS (IC_50_^1^, µg/mL)	DPPH (IC_50_, µg/mL)	FRAP (mmole FSE ^2^/g)	
FGE	421.2 ± 46.1	524.6 ± 60.9	212.0 ± 14.5	132.7 ± 0.1
Ascorbic acid	200.0 ± 0.4	128.2 ± 17.5	11,523.5 ± 35.1	–

FGE, ethanol extract of *Filipendula glaberrima* Nakai; ABTS, 2,2-azino-bis(3-ethylbenzothiazoline-6-sulfonic acid); DPPH, 2,2-diphenyl-1-picrylhydrazyl; FRAP, ferric reducing antioxidant power. ^1^ Half maximal inhibitory concentration. ^2^ Ferrous sulfate equivalent. ^3^ Gallic acid equivalent.

## Data Availability

Not applicable.

## Sample Availability

Samples of the compounds are not available from the authors.
